# Prize Essay on Cretinism

**Published:** 1860-10-01

**Authors:** 


### MEDICO-PSYCHOLOGICAL SOCIETY OF PARIS—PRIZE ESSAY ON CRETINISM.

THE Medico-Psychological Society of Paris has decided that the prize of 500fr. founded by M. Ferrus, augmented with 500fr. by M. Belhomme, and to which a member, who wishes that his name should not be known, has also added another sum of 50fr., shall be given to the author of the best essay upon "The Nature and Causes of Cretinism."

The following are the regulations which have been issued by the Society for the guidance of competitors:—

"La Société médico-psychologique demande des documents scientifiques originaux recueillis aux sources mêmes de l'observation. Ces documents devront comprendre principalement:

"1. Des topographies comparées des localités frappées et non frappées de l'endémie crétinique, soit dans la même vallée, soit dans des vallées différentes.

"Chaque topographie devra fournir des notions positives et scientifiques sur:

"1° L'altitude de la localité;

"2° La nature, la configuration, et l'exposition du sol;

"3° La nature des eaux, la composition et l'état hygrométrique de l'air atmosphérique;

"4° Le nombre, la disposition, et l'état des habitations et de leurs dépendances;

"5° L'état de l'agglomération d'habitations en tout ce qui se rapporte à l'hygiène publique;

"6° Les habitudes de la population en ce qui concerne l'hygiène privée, alimentation, vêtements, etc., etc.;

"7° La nature des occupations et de taux les salaires;

"8° La nature des relations avec les agglomérations voisines;

"9° Les coutumes en ce qui touche les mariages et l'éducation des enfants;

"10° L'état de l'instruction et la nature des institutions destinées à le développer;

"11° L'indication exacte pour chaque agglomération du nombre des habitants et des familles, et du nombre des crétins et des familles de crétins, en s'abstinant soigneusement de confondre avec les crétins les individus atteints d'idiotie simple, et en rapportant les crétins à trois groupes, suivant qu'ils sont complétement privés, plus ou moins faiblement en possession, et notablement doués de l'intelligence et de la parole;

"12° Des renseignements aussi exacts que possible sur l'histoire du développement du crétinisme dans la localité.—Le crétinisme y a-t-il existé de temps immémorial? S'y est-il manifesté pour la première fois à une époque certaine, et dans quelles conditions, par immigration de famille de crétins, par mariages?—Y a-t-il diminué et s'y est-il éteint, et sous l'influence de quelles causes, émigrations, ouvertures de routes, développement du commerce, de l'industrie, etc.?

"II. Des observations développées de familles de crétins.

"On indiquera les divers degrés de crétinisme dont chaque membre se sera trouvé atteint, et les faits d'immunité individuelle dans le plus grand nombre possible de générations.

"L'histoire de ces générations, dans leurs alliances par mariage et dans les autres conditions de leur vie, lieu d'habitation, profession, instruction, etc., devra être exposée pour le plus grand nombre possible d'individus.

"On cherchera à éclaircir, au moyen de ces observations, les points principaux de l'histoire du crétinisme, notamment ceux qui se rapportent à l'époque de l'invasion du crétinisme, soit avant, soit après la naissance; aux affinités, connexions ou dissemblances qui existent entre le développement du goître et le développement du crétinisme; à l'éducabilité, à la faculté géneratrice chez les crétins, à la prophylaxie et à la cure du crétinisme.

"III. Des observations individuelles de crétins, complétées par l'autopsie cadavérique, qui devra comprendre non-seulement une étude approfondie de tout ce qui se rapporte au volume, à la forme du crâne et de la colonne vertébrale, et à l'état de l'encéphale et de la moelle épinière, en recourant, pour donner de la précision aux faits, à la méthode de la mensuration et des pesées, mais encore des données détaillées sur l'état de tous les viscères intérieurs et de l'organisme en général.

"Les mémoires seront écrits en langue française, italienne, allemande, anglaise, espagnole ou latine.

"Ils devront porter une épigraphe qui sera reproduite dans un billet cacheté, indiquant le nom et la demeure de l'auteur.

"Les mémoires devront être adressés à la Société médico-psychologique avant le 1er juillet 1862, terme de rigueur.

"Le prix consistera en une médaille de la valeur de 1500f."

